# ‘Sussing that doctor out.’ Experiences and perspectives of people affected by hepatitis C regarding engagement with private general practitioners in South Australia: a qualitative study

**DOI:** 10.1186/s12875-017-0669-2

**Published:** 2017-11-29

**Authors:** Jane Scarborough, Emma Ruth Miller, Paul Aylward, Jaklin Eliott

**Affiliations:** 1 College of Medicine and Public Health, Flinders University, Adelaide, Australia; 2 School of Public Health, Faculty of Health and Medical Sciences, University of Adelaide, Adelaide, Australia

**Keywords:** Hepatitis C, General practitioners, Physician-patient relations, Qualitative research, Therapy

## Abstract

**Background:**

Australians with chronic hepatitis C (HCV) can access affordable Direct Acting Antiviral (DAA) treatments with high cure rates (>90%), via General Practitioners (GPs). Benefits from this treatment will be maximised if people with HCV readily disclose and engage with private GPs regarding HCV-related issues. Investigating the perceptions and experiences of people affected by HCV with GPs can allow for this pathway to care for HCV to be improved.

**Methods:**

In 2013–2014, 22 purposively sampled participants from South Australia (SA) were interviewed. They a) had contracted or were at risk of hepatitis C (*n* = 10), b) were key workers who had clients affected by HCV (*n* = 6), and c) met both a) and b) criteria (*n* = 6). The semi-structured interviews were recorded, transcribed and thematically analysed.

**Results:**

People affected by HCV viewed GPs as a source of general healthcare but, due to negative experiences and perceptions, many developed a strategy of “sussing” out doctors before engaging with and disclosing to a GP regarding HCV-related issues. Participants were doubtful about the benefits of engagement and disclosure, and did not assume that they would be provided best-practice care in a non-discriminatory, non-judgemental way. They perceived risks to confidentiality and risks of changes to the care they received from GPs upon disclosure.

**Conclusion:**

GPs may need to act in ways that counteract the perceived risks and persuade people affected by HCV of the benefits of seeking HCV-related care.

## Background

Up to 249,000 Australians, representing more than 1% of the Australian population, are estimated to be chronically infected with hepatitis C virus (HCV), with approximately 28% undiagnosed [1]. HCV transmission occurs by blood-to-blood contact and, in Australia, most commonly through illicit injecting drug use [2]. A range of symptoms is associated with chronic HCV infection which, without antiviral treatment, can lead to liver damage and, potentially, hepatocellular carcinoma [1]. Reducing the impact of HCV requires increased rates of diagnosis and increased uptake of antiviral treatment [2].

The uptake rate in Australia for antiviral treatments for HCV has dramatically improved from as few as 2% in 2012 [3] of those infected entering treatment to 14% in 2016 with advances in treatment [4]. Previous available antiviral treatments for HCV in Australia were pegylated interferon-based, involved complicated medication regimes, severe side effects, long treatment periods and variable rates of cure [5]. New direct acting antiviral (DAA) treatments for HCV have achieved viral clearance (representing a cure) in more than 90%, with reduced treatment time and few side effects [6]. From March 2016 DAA treatments approved for use in Australia were included on the pharmaceutical benefits scheme (PBS), which subsidises treatment, making these treatments affordable for Australian citizens [7].

Australian General Practitioners (GPs) are well placed, professionally and geographically [8], to be involved in HCV diagnosis, prescribing antiviral treatment and referral to specialists for HCV [9–13]. Australia provides universal access to general practice, with the Federal Government subsidising fee-for-service payments through the Medicare program [14] and approximately 80% of Australians access general practice annually [15]. Most people diagnosed with HCV in Australia are diagnosed by GPs [16] and since the introduction of the new DAA for HCV 19% [4] of those treated have had this prescribed by GPs. The majority of people with HCV are expected to be prescribed DAA for HCV by GPs [17–21] and GP involvement in prescribing is necessary due to the limited capacity of specialists to meet demand for treatment [22].

However, barriers to GPs being involved in this work have been reported [23–25] with findings indicating that, in addition to addressing system barriers, there was a need for additional capacity building for GPs. HCV education to optimally equip GPs for this role has been proposed [24–26] and this education could benefit by including a consideration of perspectives of people affected by HCV [27].

Several studies have examined the barriers and facilitators to seeking HCV-related care amongst a range of populations, various settings, and at different stages of the cascade of care [5, 28–31]. Reported barriers to treatment access include negative patient perceptions of biomedical factors such as counter indications, side effects, and poor efficacy [31] – many of which relate to older, Interferon based treatments. Although these factors may be alleviated with the new antiviral treatments for HCV, HCV-affected people need to be aware of these advances [32], and remaining barriers may deter HCV-infected people from seeking treatment.

The association with injecting drug use often results in stigma being attached to a HCV diagnosis [33, 34]. People with HCV and people who inject drugs have reported negative experiences when seeking healthcare, and their perceptions are that this is due to stigma [35–41]. Indeed, several studies have demonstrated that some GPs hold negative views of people with HCV based on the association of the virus with injecting drug use [33, 34, 42]. This can adversely affect the nature of care provided and, subsequently, the extent to which care is sought for HCV [8, 43].

People with HCV or HCV-related issues in Australia may access GPs for their healthcare [15] but for care specific to HCV-related issues to be provided, the GP needs to be aware of the patient’s HCV status or risk factors for HCV. There is no requirement for individuals to be registered with a GP or general practice in Australia [44], so patients are free to move between GPs. As no comprehensive electronic patient record system exists in Australia [44], GPs must rely on information provided by the patient. Some patients may disclose current illicit drug use to GPs to seek help for this issue [45]. Other patients may intentionally hide their current drug use from GPs to avoid stigmatisation [8, 46] or to enable ‘doctor shopping’ or ‘drug seeking’ for drugs to be used for non-medical purposes [47]. For those whose only indicator of past drug use may be HCV, disclosure of their past history is a matter of choice [39]. Where a GP is aware of a patient’s HCV status, other health issues may be prioritised by the GP or by the patient.

Although GPs have been included as participants in previous HCV studies, these studies have not focused on the perception of care provided to the diverse range of people who would present to GPs with HCV health-related issues. Our study used qualitative methodology to investigate the perspectives and experiences of people affected by HCV with private GPs in Australia and the effect that the interactions have on perceptions about engaging with GPs about HCV-related issues. The insights gained could improve people’s engagement with GPs about issues related to HCV, encourage uptake and adherence to DAA treatments, and more efficiently reduce the prevalence of this blood-borne virus in Australia.

## Methods

### Sample and recruitment

**Fig. 1:**
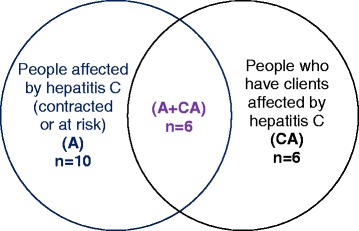
Final Sample

We used a purposive sampling strategy to gain a range of perspectives regarding individuals who potentially access HCV care in private general practice in South Australia (SA). Three groups were recruited (see Fig. 1). The first included people either at risk for, or self-reporting, a diagnosis of HCV (identified as “A”: affected by HCV). This included participants with a history of illicit injecting drug use but not currently diagnosed with HCV, who are at risk of becoming infected, or may be amongst the 20% of Australians with chronic infection who remain undiagnosed [2]. People affected by HCV in this way can provide valuable insights into their perspectives and experiences about the provision of care by private GPs.

The second group comprised key workers providing care to clients affected by HCV (identified as “CA”: clients affected) including clinicians and peer workers; these individuals can provide valuable insights about care sought and provided on the basis of their long engagement with, and advocacy for, clients affected by HCV. These two groups: “affected by HCV” and the “clients affected” were not discrete, however, as some key workers reported personal experience of a diagnosis of HCV and illicit injection drug use histories (identified as “A+CA”: affected by HCV, with clients affected by HCV). These participants were able to give perspectives based upon their own, and their clients' experiences. Purposive sampling further aimed to include participants varying in drug use status, awareness of HCV, duration of infection, and treatment status.

Recruitment occurred in three phases and primarily through two organisations, Drug and Alcohol Services of South Australia (DASSA) and Hepatitis SA. DASSA is an agency of the SA Health Department with clinics offering a variety of services including clean needle provision, opioid substitution therapy (OST) and drug counselling. Hepatitis SA is a community organisation that provides information and advocacy services, including peer education, for people affected by viral hepatitis. Recruitment of key workers from DASSA and Hepatitis SA occurred through targeted emails, as well as researcher attendance at staff meetings. Key workers targeted for recruitment included medical practitioners, nurses, social workers, psychologists, and peer educators. To recruit people affected by HCV, an advertisement was placed in the quarterly Hepatitis SA newsletter, distributed throughout SA in both hardcopy and digital form. Recruitment targeting people who had a history of injecting drug use occurred through DASSA clinics via the placement of posters and information packs, and subsequent personal approaches at the clinics. Eligible participants were older than 18 years and able to participate in the interview in English. Signed informed consent was obtained before commencement of the interview.

### Participants

Twenty-two participants were recruited (11 female, 11 male), 10 people affected by HCV (A), 6 people who have clients affected by HCV (CA), and 6 who were both affected by HCV and had clients affected by HCV (A+ CA). Other than indicating that they met the inclusion criteria of being over 18 years of age, participants were not asked to supply demographic details, but some demographic information was provided during the interviews. Participant responses indicated an age range from early 20s to over 60. Most participants were residents of Adelaide (the capitol of SA) metropolitan suburbs with only one participant (CA) residing in a regional area and one participant (A) previously residing in a regional area. Participants with clients affected by HCV provided services to HCV-affected clients across SA. One participant indicated a non-English speaking, 1st generation migrant background. No participants identified to the interviewer as an Indigenous Australian.

All participants reporting a diagnosis of HCV also reported a history of illicit injecting drug use, and that HCV transmission occurred via this practice, with the exception of one participant who reported that transmission was via non-sterile tattooing. The significance of this self-reported illicit drug use of participants varied in regard to currency (from recent to historical) and severity (chronic and relapsing, to occasional and controlled). All affected participants indicated that they had the choice to access private GPs, with some participants being currently highly engaged. Participants from the groups affected by HCV, self-reported a range of HCV diagnosis including current chronic HCV infection, intention to be tested, previous HCV infection (naturally cleared), and cured of chronic HCV infection. Participants from the groups affected by HCV also self-reported a range of involvement in HCV treatment: current, seeking treatment, successful and unsuccessful completion of treatment, decided against interferon-based treatment available at the time of the study.

### Data collection and analysis

Semi-structured qualitative interviews were conducted from November 2013 and August 2014, at a time when DAA treatment in Australia was being clinically trialled but yet to be approved for broader use or included on the PBS. Interviews were carried in a range of settings convenient to the participants, including DASSA sites, public library spaces and a participant’s home. Interview outlines were informed by the existing literature [5, 28–31] and included questions about HCV knowledge (‘Tell me how you gained your knowledge about hepatitis C’), experience of HCV (‘Can you tell me the effect of hepatitis C on your life?’), perceptions of private GPs (‘What for you makes a good private general practitioner?) and experience of care provided by GPs (‘Can you describe any situations where you have received medical care from private GPs that relates to hepatitis C?’). Interviews ranged in length from 19 min to 87 min with the median length of 58 min. The interview outline was covered during all interviews, although one participant requested the interview was kept brief. Participants were given a $20 supermarket gift voucher as compensation for their time.

Interview audio recordings were transcribed verbatim as soon as possible after the interview took place. The transcripts were de-identified with participant names replaced with pseudonyms and the text entered into NVivo 10 software [48]. Transcriptions were thematically analysed in the process outlined by Braun and Clarke [49] to identify, analyse, and report on participants’ perspectives of care provided by GPs for HCV. The interview text was then systematically coded, with some codes reflecting the literature-informed interview topics and others reflecting additional aspects of the experiences and perspectives of participants. The codes produced were then initially grouped to identify possible themes. The themes were reviewed and discussed amongst the authors to define and name the final themes reported. Pseudonyms used during the analysis were removed from the manuscript to protect participant confidentiality.

### Reflexivity

The first author works at clinical sites offering treatment for drug issues and in a role that involves interacting with a variety of private GPs. Other members of the research team were from a variety of professional backgrounds, and provided an ‘outsider’ perspective, which promoted a more reflexive analysis of the data Interpretation was enhanced through collective discussion and reflection upon emerging themes identified by the first author. This enabled different lenses to be applied and interpretive meaning across the data set to be agreed consensually. The possible effect of any one individual’s bias was therefore ameliorated by having input from the four authors throughout the research process [49].

### Ethics approval and considerations

Ethics approval was obtained from the South Australian Health Human Research Ethics Committee, the Aboriginal Health Research Ethics Committee (a sub-committee of the Aboriginal Health Council of South Australia Inc.), and the Human Research Ethics Committee of the University of Adelaide.

The first author conducted the interviews and works at DASSA in a non-clinical role. To avoid any potential ambiguity, [50] the interviewer explained her role as researcher and her separate role at DASSA, and emphasized processes to be used to protect confidentiality. Participant responses indicated that they understood choice regarding participation and that participation would not affect their ongoing relationships. The drug use and HCV status of researchers has been presented as important aspect of prior research in this field [51]. The interviewer has no history of illicit drug use or HCV and was careful to present as having no such history, allowing the participants’ decisions to be involved in the research and share information to be based on informed consent.

## Results

The experiences and perceptions of participants regarding engagement of people with HCV with GPs will be outlined. Similar experiences and perceptions were provided by participants independent of the basis of their recruitment as people directly affected by HCV (A), had clients affected by HCV (CA) or could provide both perspectives (C + CA).

### Engagement about HCV – Disclosure choices and strategies

Participants viewed GPs as a source of general healthcare that potentially includes care for issues related to HCV. Several participants stated that when they were ‘lucky’ to find a good doctor, they would attempt to continue to engage with them.

My doctor’s interested in what’s happening, asks me the right questions. Asks me what testing I’d liked to be done. Won’t write “hep C” on a referral to anyone else, without my express consent. (A)

Some participants held that the benefit of engaging with GPs could only be fully realised if they disclosed their HCV positive status and/or drug use history, placed their trust in the GPs professionalism, and were accepting of the care they received.

You know if I’m sick I’m going to have to say “Might be because …”. I want to go to find out; go to every aspect of why I might be sick. (A)

Where they perceived a lack of benefit associated with disclosure and a risk of consequences in disclosure (either within the patient–doctor relationship, or in their wider lives), people with HCV were cautious about disclosing HCV. A process of judging or ‘sussing’ out the GP before disclosing was repeatedly described.

So I think it would an issue of the client sussing that doctor out and thinking “No I don’t think that they could deal with that,” and I think they’re pretty good at that. (CA)

I can tell what kind of character he is you know. He would be one of those that just, you could just see that. He would go out of his way, but as for other doctors I’m not real sure about that you know. (A)

… clients will just go to drop in, bulk-billing places at times when they need something, and then they [the client] may link with a GP if they [the client] find they got on well with the person ….(CA)

Often participants outlined that they or their clients adopting a strategy of having one GP that they engage with for HCV-related issues, but maintaining non-disclosure with other GPs. Engagement with a nurse trained to support people with HCV to gain appropriate care in a shared care arrangement was also regarded as an effective strategy to maximise care for HCV.

I go between a couple of GPs. I also go out and see [name of doctor] out at [surgery that has blood borne virus treatment specialty] and so yeah. He’s a lot more reasonable. (A)

There’s a hep C place over at the [name of practice] over there and that’s where my nurse is. You know they’re pretty supportive there. (A)

### Perception of engagement involving risk to confidentiality of sensitive information

Engagement with GPs that involved disclosure of HCV positive status was seen by some participants as a risk to the confidentiality of this sensitive information, with potential serious consequences. Some participants had not disclosed their HCV status to their work, families, or their intimate partners. They reflected that their illicit drug use was part of their past and that they should be in control of the decision to disclose any of this information. These participants said that they were not suspected of a past drug history and that this history would remain secure if they managed disclosure about HCV.

Well I still haven’t told my parents. (A+CA)

… my partner doesn’t know and I’ll never tell him. I don’t think it’s relevant. (A+CA)

Possible breaches of confidentiality by medical practitioners providing HCV-related care were seen to diminish the non-disclosure choice of people affected by HCV. Participants described people affected by HCV as being ‘on constant guard’ to protect this information. The need for a person’s HCV status to be shared between healthcare workers was questioned, as the participants expected that all health practitioners should use universal precautions to avoid transmission, and considered that this would negate the need to share this information in most circumstances. Where breaches of confidentiality had occurred, this led affected participants to question whether the health practitioners involved in their care understood the significance of their choice to limit the disclosure of their HCV positive status.

… my kids got asked “Could you get your mum to ring the hospital urgently?” and I’d made it really clear I have not told anybody in my family about this, do not leave messages. (A+CA)

I used to tell them [healthcare workers] because “it’s my responsibility of blood you know, infectious blood” and all that sort of thing. And then in at the [name of hepatitis C support organisation] they said “Well actually it is their responsibility. You don’t have to tell anybody.” (A)

HCV-affected participants reported feeling discomfort when their HCV status was recorded on patient records. They felt that the standard practices associated with data collection and patient records presented a risk to confidentiality and control of information. Participants proposed that engagement with GPs was affected by these concerns and that they, or their clients, had not disclosed information, and indeed, avoided returning to practices where they had reluctantly disclosed.

Every worker can look up my records. Do I like that? No I hate that with a passion. (A+CA)

… this form, this is really bad; this form goes to the receptionist and the receptionist keeps it there, puts whatever she wants, [it] sits on the receptionist’s desk and then it gets given back to and given back to the doctor. So this form is floating all around the place … (A)

The understanding of the sharing of information was informed by participants’ involvement in highly regulated opioid substitution programs. One participant perceived that this healthcare was only available on the condition that they subjected themselves to the rules of the program, including being tested for HCV, stating “I’ve got to do a blood test soon” (A), and that information would be electronically shared between the program and GPs, as “It’s all linked” (A).

### Perception of engagement involving risk of exposure to discrimination, negative judgement and change to care provided.

Based on their expectations and experiences, participants did not assume that all GPs would be willing to provide care for HCV-related issues in a non-discriminatory, non-judgemental way. Participants stated they understood that GPs could develop negative attitudes to people with a drug history due to being exposed to ‘drug seeking’ or ‘doctor shopping’ behaviour, and categorised this reaction by GPs as somewhat reasonable. The majority of participants detailed how they or their clients had participated in these behaviours to obtain drugs to be used for non-medical purposes.

But you can’t blame the GPs being like they are because you’ve got no idea what some people do to get drugs out of them. (A)

Depending on the doctor you could pretty much talk your way into getting anything you want, but if you do want something in the future and you do actually need it, they’re not going to give it to you if they’ve found out you’ve lied ….(A)

Two participants described GP practices as ‘banning’ a patient in response to patient bad behaviour and an expectation that if a patient was suspected of ‘doctor shopping’ or ‘drug seeking,’ they may be banned. For one patient, such a ban meant that there was no primary health care available in the local area.

…of course when they [patients] act up then they’ve got bans [from attending GP practices] and things like that, so yes there are issues…. I’ve got one client in [neighbouring rural town] that has been banned from every clinic in [major rural centre] plus [neighbouring rural town within travelling distance for client]. (CA)

One participant reported their perception there was a hurdle of stigmatisation to overcome when telling a GP about drug use, which, as a necessary step to getting help, meant that help was therefore not assured. This participant was affected by HCV and described approaching GPs for help for drug issues as part of the process of engaging with them regarding HCV.

You don’t tell a doctor “I’m using drugs. Don’t help me please.” It’s a cry out for help isn’t it? You know. Like it means if you get ignored you’re like “What did I bother telling them for?” Especially when it’s such a frowned upon thing. (A)

Whilst overt discrimination by GPs based on HCV status was not reported, a perception of subtle changes to GP behaviour upon disclosure of HCV status was.

Yeah just her [the GP’s] whole attitude like really changed towards, you know there wasn’t that kind conversation anymore, yeah you know. (A)

Participants reported exposure to discrimination in other healthcare settings and this exposure appeared to be incorporated into their perception of expected risk, and increased their expectation, of discrimination from GPs.

… and he [surgeon] said “I see you’ve had treatment.” And I said “Yeah but it didn’t work.” And it was like he was on a rubber band, he flew back to the back of his chair. … and then he came up with all these excuses why I couldn’t have this operation. (A)

Participants also described experiencing a shift of attributed identity upon disclosure—from ‘patient’ to ‘drug-addicted patient,’ with an associated change to the care received. For instance, participants related that they were prescribed drugs of dependence when their HCV status was unknown, but that GPs would become extremely reluctant to prescribe these drugs once aware of their HCV status. Some participants framed their past illicit drug use as something that they had left behind and as not requiring treatment. Offers by GPs for drug treatment were considered by these participants as well-intentioned, but misplaced and irrelevant.

If I said you know, had injected drugs in the past they would always enquire whether I needed ‘rehabilitation’ and it was if ever I needed any pain relief, I broke a rib once and the doctor said “I can’t give you anything stronger than Panadol unless I send you off for an X-ray.” (A+CA)

Some HCV-positive participants expressed fear of having important aspects of their lives judged by GPs and other healthcare workers. In particular, participants expressed concern that their decision to have children would be negatively judged due to the children being ‘exposed’ to the risk of transmission through household or in-utero transmission.

… I’ve heard some horror stories of what people have you know experienced with GPs and hospitals, … there’s even this feeling that you know you don’t deserve to have children because you know the risk that you’re putting them through ….(A)

### Uncertainty about the benefit of engaging with GPs about HCV – Based on perceived misinformation provided and information gaps from GPs

Participants described that they and other people with HCV had developed knowledge about HCV, sometimes over the long duration since diagnosis. They proposed that their HCV knowledge could sometimes exceed that expected of their GPs: “I … found out what I could and what I needed to know” (A). The difficulty of GPs keeping abreast of all of the detail of the conditions that they encounter was often acknowledged by participants.

… hep C [is] not fully understood by GPs and I’m not blaming them for that, just ‘cause they’re trying to grapple with so many attends [sic], that’s impossible. (A)

Some participants also acknowledged that they had misunderstood what they had been told: “I didn’t understand I had hep C” (A + CA).

Most participants reported that HCV-affected people had accumulated experiences of being provided what they perceived to be inadequate or inaccurate information by GPs in regard to HCV. This perception contributed to a lack of certainty by participants that GPs would provide accurate, up-to-date information regarding HCV. Some participants spoke of errors by some GPs, which they attributed to GPs limited knowledge about HCV testing, sometimes leading to serious repercussions for patients. A participant stated one of their clients was given a diagnosis of HCV, which was subsequently found to be incorrect. Another stated that they had been told that they had cleared the virus when they had not.

… she believed she had hep C for over 12 years and was in [rural town] and the GP had never done a PCR test [polymerase chain reaction test that is need to confirm active HCV infection]. (A+CA)

… I was told that my bloods functions were back to normal and that I had cleared the virus. Not knowing any different I believed it ….(A)

Some participants affected by HCV articulated previous gaps in their knowledge about transmission, and suggested that GPs had missed opportunities during consultations to provide such important information. For example, it was reported that GPs did not provide safe-injecting information to those patients they knew injected illicit drugs, or provide information about household transmission to people with a diagnosis of HCV.

Just didn’t tell me how it was passed on or anything like that. I’d been seeing this guy [GP] on and off for 20 years so he know [sic] all my background and everything anyway so I suppose he didn’t even think to mention anything to me ….(A)

Some HCV–affected participants stated that they had an incorrectly reduced perception of harms associated with HCV based on what a GP had told them, or from the GP’s lack of follow-up or encouragement to enter treatment. Some participants expressed unease that clients were not appropriately monitored by GPs, with blood tests or liver scans, for example.

I took the doctor’s on their word and pretty much didn’t worry about it. (A+CA)

… doctors are telling people that you know that they [alanine aminotransferase levels which indicates liver damage or disease] might be slightly elevated but “that’s OK so don’t worry about it,” so they’re not actually giving them information. So as long as you’re not experiencing symptoms then it’s OK. (A+CA)

I am aware that some GPs never do bloods and they don’t seem to monitor people that even if they know that clients got hep C ….(CA)

Several participants who had delayed antiviral treatment as a consequence of a perceived lack of GP concern stated their specialists had indicated that the delay had caused negative effects on their health by the time they commenced treatment.

I did ask doctors that question “Is that OK?” and they would say “Yes” now the specialists say “No mate you should have had this years ago.” (A+CA)

### Perception of benefit: Engagement to access antiviral treatment

This study was conducted whilst the new DAA treatments were in the trial phase and several participants who were affected by HCV spoke of the potential of new treatments to have fewer side-effects, contributing to their decision to delay consideration of treatment. Participants indicated that they would revisit their decisions regarding antiviral treatment when the new treatment was available.

They [specialist at tertiary hospital] said that in the future there’d be medication coming out but at the moment I can’t get this [interferon-based] medication because it’s [HCV] not really bad yeah. (A)

Obtaining a referral for treatment by a specialist (required for previous interferon-based treatment) was the only rationale provided by many participants for engagement with a GP about HCV-related issues. Accordingly, patients deciding against this treatment at the time did not consider any benefit from engagement with GPs about HCV.

I mean the only basic help like that that they [GPs] could do for you is, you know, is write you out a referral to somewhere else but I’ve never seeked [sic] help before for my liver problem because of that reason. (A)

Negative perceptions and experiences about the physical side-effects of treatment were relayed by participants and some had decided that treatment was not currently an option for them.

One of my friends went on that thing [antiviral treatment for HCV] to get rid of it but she got really sick from it ….Yeah it dehydrated her brain; she nearly died ….(A)

One participant reported that their GP held negative views of the available, older interferon-based, antiviral treatment and this had led to him refusing to make a referral for a HCV-related investigation or for antiviral treatment: “he was quite clear that I shouldn’t have that …” (A).

Finally, some participants who had sought HCV antiviral treatment at tertiary centres cited situations in which the treatment had not been provided, despite their GP providing them with the required referral and even where the GP had actively followed up the progress of the referral. They recognised that the lack of treatment provision was the responsibility of the tertiary centre and not of the GP, but for these participants, engagement with GPs was perceived as an ineffectual pathway to their goal of antiviral treatment for HCV.

But he’s [the participant’s GP] put referrals in through the [tertiary hospital #1] but I don’t hear anything about the referrals, ay. (A)

… you get an appointment and then they [tertiary liver clinic] pretty much give you a blood test and then they say “come back in six months.” (A)

## Discussion

Since this study was conducted, affordable, direct acting, antiviral treatment for HCV has become available for Australian citizens [7] and this represents a spectacular advance in the potential to address the biomedical aspects of HCV infection [6]. Most Australians (including all participants) access private, federally-subsidised GPs for their healthcare [15] and,for the majority of people with HCV in Australia, this relatively new treatment can be delivered via private GPs [13]. For people to access this treatment, however, they need to disclose their HCV status or HCV risk factors and be willing to engage with GPs about this issue. The aim of this study was to better understand the experiences and perceptions of people affected by HCV regarding engagement between GPs and patients about issues relevant to HCV. This information could be used to guide the development of approaches for private GPs to reduce barriers to, and improve the delivery of, care for people with HCV.

Like the general Australian community, people affected by HCV viewed GPs as a source of general healthcare [13], potentially including issues related to HCV. Several participants reported being highly satisfied with the care provided by individual GPs including specific care for their HCV. This notwithstanding, they and other participants also relayed negative experiences and perceptions about engagement with GPs for HCV-related care. Consequently, participants often described people affected by HCV developing a strategy of “sussing” out doctors before engaging and disclosing to individual GPs. The process involved weighing up the perceived risks and the perceived benefits and then deciding whether engagement and disclosure were worthwhile. As reported in previous studies [35–40], many of these risks were related to stigma, but several other factors also contributed to the decision-making process. If GPs are aware of the decision-making process that people affected by HCV undertake, the GPs can utilise strategies aimed at reducing their doubts and increasing their perceptions of benefit about the care they will receive, and tip patients’ decisions towards disclosure and engagement.

Participants acknowledged the difficulties for GPs dealing with ‘doctor shopping’ and other ‘drug seeking’ behaviour and, particularly where they had previously participated in it, expressed sympathy for doctors required to deal with such behaviours. The honest disclosure of current illicit drug use to a GP, however, would be illogical if the person was attempting to obtain drugs of dependence without genuine need. This is more likely to represent an attempt to elicit care for HCV, or a health issue related to drug use. Faced with this situation, GPs might offer people a range of information to prevent harm, for example, brochures regarding prevention of transmission of blood-borne viruses. In addition to the benefits for patients receiving opioid substitution treatment [52, 53], GPs may find that drug-seeking behaviour is reduced when they offer such treatment [45].

Based on their expectations and experiences participants did not assume that all GPs would be willing to provide care for HCV-related issues in a non-discriminatory, non-judgemental way. Importantly, no participant described overt discrimination associated with HCV by GPs. Discrimination in other settings, including health settings, was described, and these experiences appeared incorporated into participants’ expectation of encountering discrimination from GPs. Displaying posters and other information in the waiting room about hepatitis C or drug use can signal to patients that the practice is willing to provide care for these conditions. This material would be timely as our participants’ responses indicate that when the new DAA therapies were available, people affected by HCV would reassess their previous decision to not enter treatment. Additionally, publicity surrounding these new treatments may prompt people who are undiagnosed to consider their risk of exposure, and the presence of this material in the waiting room may prompt them to seek testing at the practice.

Upon disclosure of an HCV diagnosis to GPs, some participants recounted experiences of unwelcome changes to their usual care, which precipitated reluctance to disclose to other practitioners in the future. Unwillingness to prescribe drugs of dependence, and focussing on the patient’s ‘drug-use problem’ were reported by participants as examples of GPs’ reactions following disclosure of their HCV-status. Participants expressed a frustration that, whilst well intentioned, this change appeared to reflect GPs’ incorrect assumptions concerning ‘inherent’ relationships between HCV and ‘current’ drug addiction, when in fact there is a great deal of heterogeneity of the population affected by HCV regarding such behaviours or identities [2, 29, 52]. GPs can play an important part in addressing patients’ drug-use issues [54] but should not assume that all patients presenting for HCV management require treatment for drug use. Whilst drug addiction is often described as a ‘chronic relapsing disorder’ [55], not all people with HCV are, or have ever been, addicted to drugs [2, 29, 52]. It is good practice for GPs to consistently exercise caution prescribing drugs of dependence [55], but the use of these drugs are warranted to treat many conditions. In these circumstances, GPs should not deny drugs of dependence to patients, based on the patient’s HCV status or even reported drug issues, but should exercise clinical judgement using appropriate safeguards when prescribing [55].

A source of concern for some participants was the loss of control of their information, with the subsequent risk to the confidentiality of sensitive information and potential associated serious consequences. Open disclosure within the patient-doctor relationship did not equate with disclosure to family, intimate partners, friends, or during employment. These participants wanted to avoid exposure to stigma due to their HCV status and their past injecting drug behaviour if this became known [31, 39, 56]. Australian general practice standards [57] require practices to adhere to policies regarding the protection of patient information. Routinely informing all patients that these systems are in place, would reduce fear, and encourage open and honest disclosure, about sensitive information, in particular drug-related or HCV-related conditions. Participants wanted to be reassured by their GP that their information would only be shared with their explicit permission, and only when relevant. Even when confidentiality policies are strictly adhered to, concerns about privacy may still be held by the patient. Being provided reassurance about this aspect of care is an important way to address concerns and improve engagement.

Participants recognised the difficulty of GPs maintaining in-depth knowledge over the wide scope of their work, yet, as reported elsewhere [58], it was common for participants to be critical of the information provided by some GPs regarding HCV. It is unsurprising that participants had a high level of knowledge underpinning this judgement as many had actively accumulated knowledge since receiving their HCV diagnosis, had health related qualifications, and/or worked with clients affected by HCV. It is important for GPs to be able to demonstrate up-to-date knowledge about HCV diagnosis, management, and treatment to convince people affected by HCV to engage with them. The DAA treatment for HCV is relatively simple compared to former treatments and its availability provides a trigger for GPs to be recruited into education to bridge GPs’ identified HCV knowledge gaps [24]. Education must take into account the time restraints of GPs and incorporate the patient perspective to barriers to treatment. Where GPs undertake education and training, it would be useful for people seeking care to be aware of this through a register and/or signage at GP practices.

All people affected by HCV should have access to GPs providing best practice care for HCV-related issues. This study has helped to illuminate how the perceptions of people affected by HCV influences and defines for them the nature of their engagement with GPs around HCV care. However, further research could usefully explore the nature of meanings and perceptions brought to the potential consultation by GPs, which may enhance or limit the extent to which they are able to appropriately and successfully engage with these patients and address the concerns raised in this paper. This may also allow the development of practices and policies that better address situations where HCV affected patients appear to have been effectively “banned” from certain South Australian practices.

The lack of participants who identify as Indigenous Australians is a limitation to this study. Further research with Indigenous Australian participants is necessary to determine if the findings are applicable to this population, to identify any additional beliefs or practices that may contribute to their perspectives and experiences, and to outline the implications of same for best practice care [59]. A further potential limitation of this study is that the findings in one Australian state (SA) may not be generalizable to jurisdictions where different systems of primary medical care exist and other dynamics are present. Experiences of stigma have, however, been reported internationally [31] and our findings will be relevant wherever HCV-affected people face decisions about managing disclosure and engagement when seeking primary healthcare. This study was conducted before DAA treatments for HCV options became widely available in Australia, and this forms another potential limitation. Although participants indicated that they would reassess entering treatment when new DAA treatments became available, people would need to be assured that they could access this treatment via GPs; some may not engage and disclose if they perceive the associated risks to be too high. Understanding the patient decision-making process regarding engagement with GPs will therefore still be relevant in the era of DAA HCV treatment availability.

## Conclusion

People affected by HCV come to any GP-patient interaction with a background of experiences and perceptions, all of which shape their expectations of the benefits and risks of engagement. Capable and willing GPs may need to act to counteract the perceived risks and persuade those people “sussing them out” of the benefits of seeking HCV-related care from them.

To do this, GPs can publicise that they are willing and able to provide care for HCV and related conditions, including making available information about the new DAA treatment for HCV and its benefits. Stating and demonstrating adherence to the confidentiality policy of their practice will reassure patients that they are in control of their information and that disclosing to the GP will not increase the risk of exposure to stigma. Each patient comes to the patient-doctor relationship with their own history, understandings, and set of needs. GPs can offer appropriate individualised care to patients affected by HCV by assessing each individual patient’s situation and requirements.

## Abbreviations

DAA: Direct Acting Antivirals
DASSA: Drug and Alcohol Services South Australia
GPs: General Practitioners
HCV: Chronic hepatitis C
PBS: Pharmaceutical benefits scheme
SA: South Australia
